# Case report: A novel PTCH1 frameshift mutation leading to nevoid basal cell carcinoma syndrome

**DOI:** 10.3389/fmed.2024.1327505

**Published:** 2024-03-04

**Authors:** Xiaoqing Lang, Ting Wang, Shuping Guo, Yao Dang, Yingjie Zhang, Hongye Liu, Hongxia He, Li Li, Huajie Yuan, Ting He, Qiong Wang, Shiyu Qin, Runping Cheng, Xingquan Yan, Hongzhou Cui

**Affiliations:** ^1^Department of Dermatology, First Hospital of Shanxi Medical University, Taiyuan, China; ^2^Department of Dermatology, Shanxi Provincial Integrated Traditional Chinese Medicine and Western Medicine Hospital, Taiyuan, China; ^3^Department of Nursing, Fenyang College of Shanxi Medical University, Fenyang, China

**Keywords:** novel, frameshift mutation, nevoid basal cell carcinoma syndrome, PTCH1, case report

## Abstract

A patient presenting with several basal cell carcinomas, pigmented nevi, and developmental defects was diagnosed with nevoid basal cell carcinoma syndrome. Gene panel sequencing and Sanger sequencing were used to identify a novel heterozygous frameshift mutation, c.1312dupA:p.Ser438Lysfs, in exon 9 of PTCH1. I-Tasser and PyMol analyses indicated that the mutated protein patched homolog 1 (PTCH1) lacked 12 transmembrane domains and the intracellular and extracellular rings of ECD2 compared with the wild-type protein, resulting in a remarkably different structure from that of the wild-type protein. This case extends our knowledge of the mutation spectrum of NBCCS.

## Introduction

Nevoid basal cell carcinoma syndrome (NBCCS; OMIM 109400), also known as Gorlin syndrome, is a rare autosomal dominant disorder with multisystem involvement. The approximate prevalence is one case per 56,000–164,000 population. The characteristic features of the disorder are numerous basal cell carcinomas, odontogenic keratocysts of the jaw, and developmental defects such as vertebra/bifid ribs, intracranial calcification, and polydactyly. NBCCS also predisposes some patients to various low-frequency tumors, such as ovarian fibroma, medulloblastoma, rhabdomyosarcomas, and cardiac fibromas (1). Ocular anomalies occur in some cases, including strabismus, congenital cataracts, eyelid cysts, myelinated nerve fibers, and pigmentary changes of the retinal epithelium (2). Multiple organ systems may be affected in NBCCS. The most common abnormalities involve the skin, the skeletal system, the genitourinary system, and the central nervous system. The disease severely affects the quality of life of patients and has a high disability rate, which is mainly caused by mutations in the PTCH1, PTCH2, or SUFU genes (3). Most NBCCS patients have pathogenic variants of the PTCH1 gene, whereas PTCH2 and SUFU mutations are less common (4). Herein, we report the case of a 56-year-old woman with typical clinical and histologic findings of NBCCS and a frameshift mutation in the PTCH1 gene.

## Case presentation

**Figure 1:**
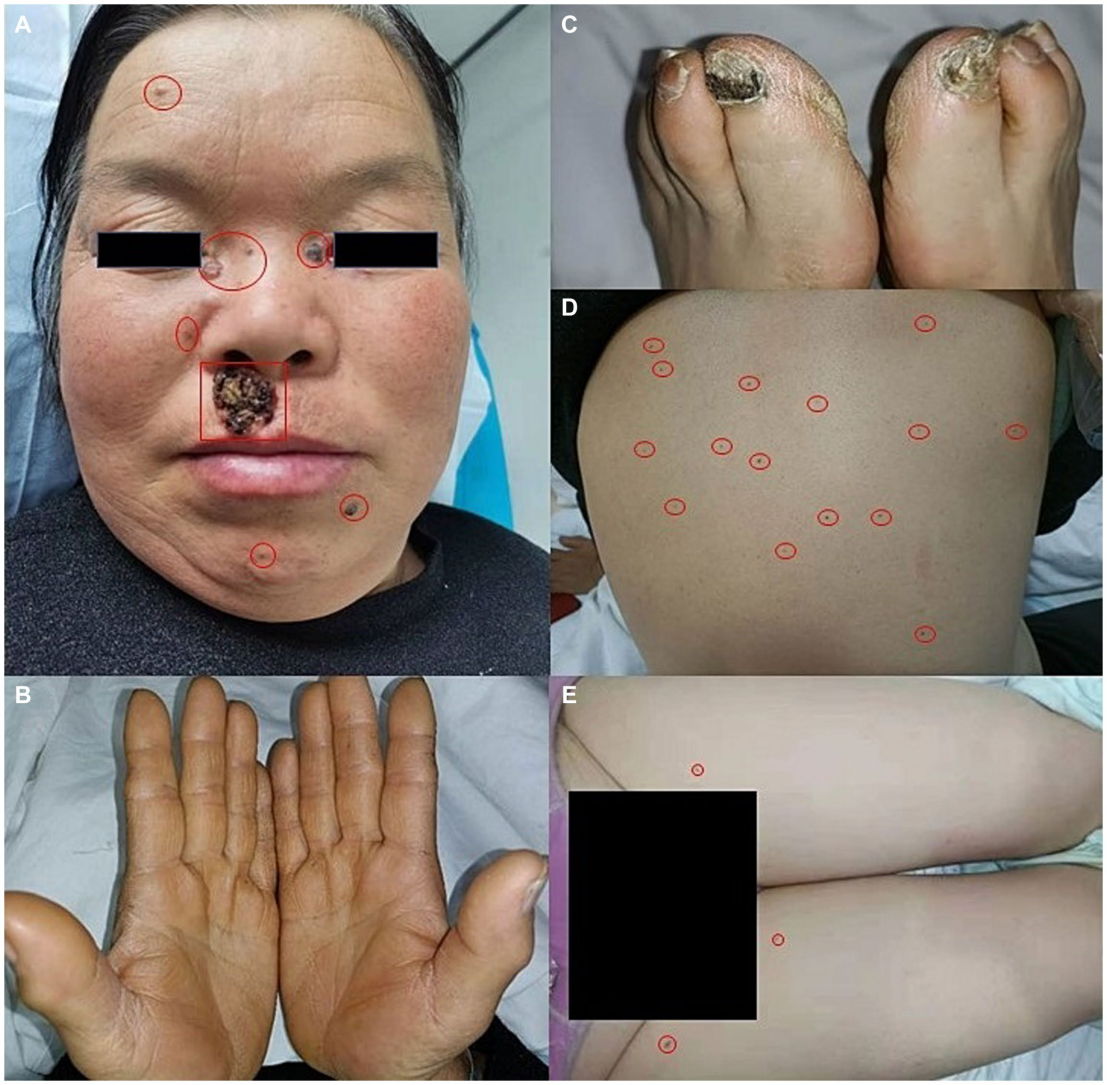
**(A)** Dark brown papules and plates on the face, broad confluent eyebrows, a broad base of the nose, and frontal bossing. **(B,C)** Dry, rough, and keratinized skin on both hands and feet, dark brown great toenails on bilateral feet. **(D,E)** Multiple pigmented nevi on the back and thighs.

**Figure 2:**
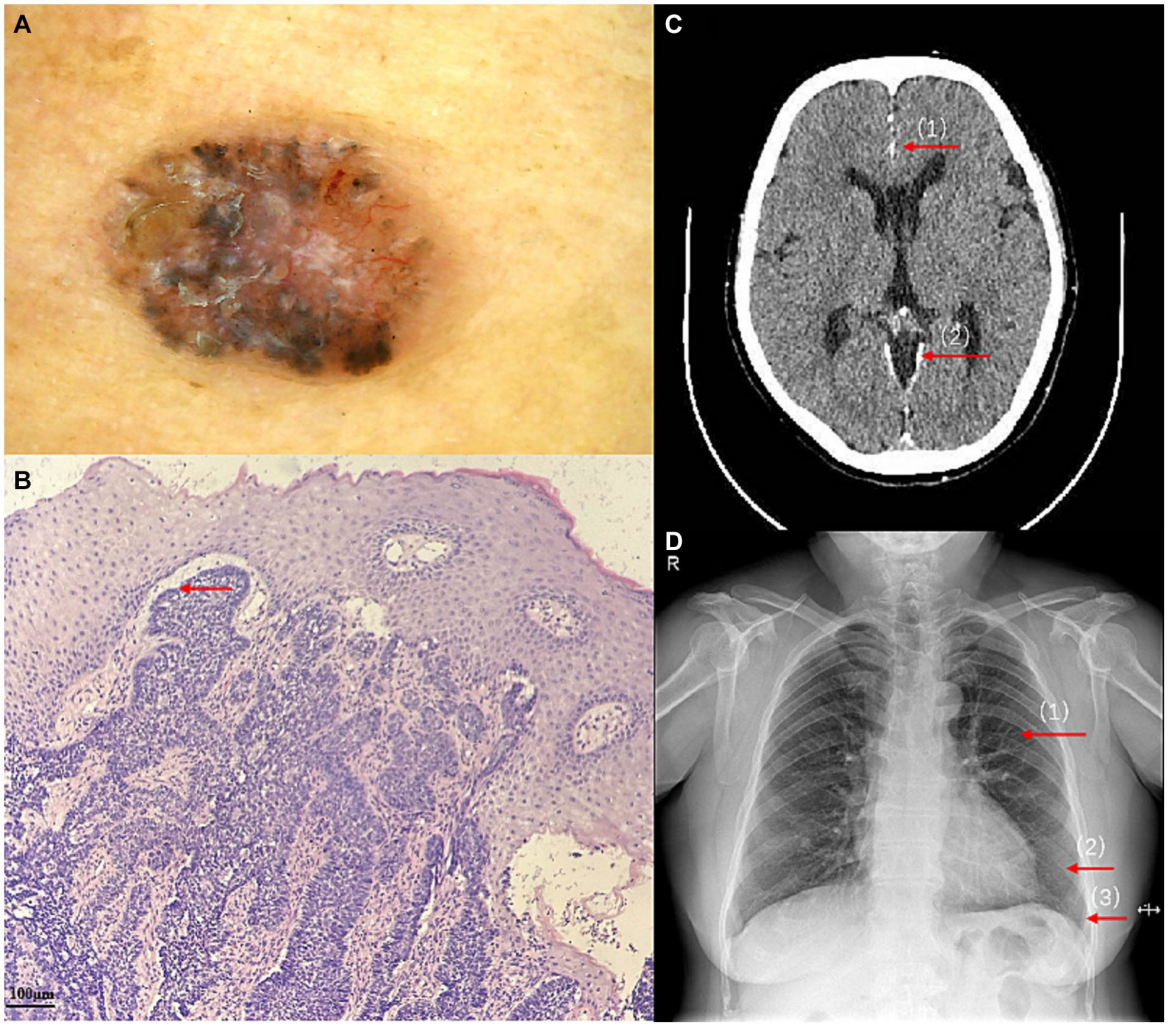
**(A)** Dermoscopy at a 50-fold magnification showed many irregularly distributed blue-gray spots and small balls, bright white stripes, oval nests, and linear branching of blood vessels. **(B)** The proliferation of basaloid cells and retraction artifacts (HE ×100). **(C)** Calcification of the falx cerebri (1) and tentorium cerebelli (2), and mega cisterna magna. **(D)** bifid thoracic ribs:Second (1), sixth (2), and seventh (3) anterior ribs on the left side.

A 56-year-old woman presented to our dermatological department with dark brown papules and plates measuring 1–3 cm in diameter and located on the patient’s face, some of which showed a broken and scabbed surface (Figure 1A). The skin of both hands and feet was dry, rough, and showed keratosis, and the toenails of both feet were dark brown (Figures 1B,C). Multiple pigmented nevi were observed on the back and thighs (Figures 1D,E). The patient had broad confluent eyebrows, a broad base of the nose, and frontal bossing. Dermoscopy showed many irregularly distributed blue-gray spots and small balls, bright white stripes, oval nests, and linear branching of blood vessels (Figure 2A). Histopathological examination of the four skin areas on the face showed nests of tumor cells in the dermis. The nests were characterized by proliferation of basaloid cells with palisading nuclei at the periphery of the nests. The loose tumor stroma showed retraction artifacts (Figure 2B). Radiological examination revealed calcification of the falx cerebri and tentorium cerebelli, scoliosis, bifid thoracic ribs, and mega cisterna magna (Figures 2C,D). The cardiac ultrasound and gynecological ultrasound showed no abnormal findings.

Three years before this presentation, the patient received treatment for multiple jaw cysts in the Department of Stomatology, the First Hospital of Shanxi Medical University, Taiyuan, China. Histopathological examination of the jaw cysts showed keratocysts lined with stratified squamous epithelium that lacked a granular cell layer. The patient’s son and second daughter had wide eyebrows, wide nasal roots, and prominent foreheads. They had multiple jaw cysts and underwent surgical treatment in our hospital. Postoperative pathology showed keratocysts lined with layered squamous epithelium that lacked a granular cell layer. In addition, the patient’s mother died of a tumor, and her sister was diagnosed with a nasal tumor in our provincial cancer hospital. Her first daughter died of rectal cancer 1 year prior to this study.

**Figure 3:**
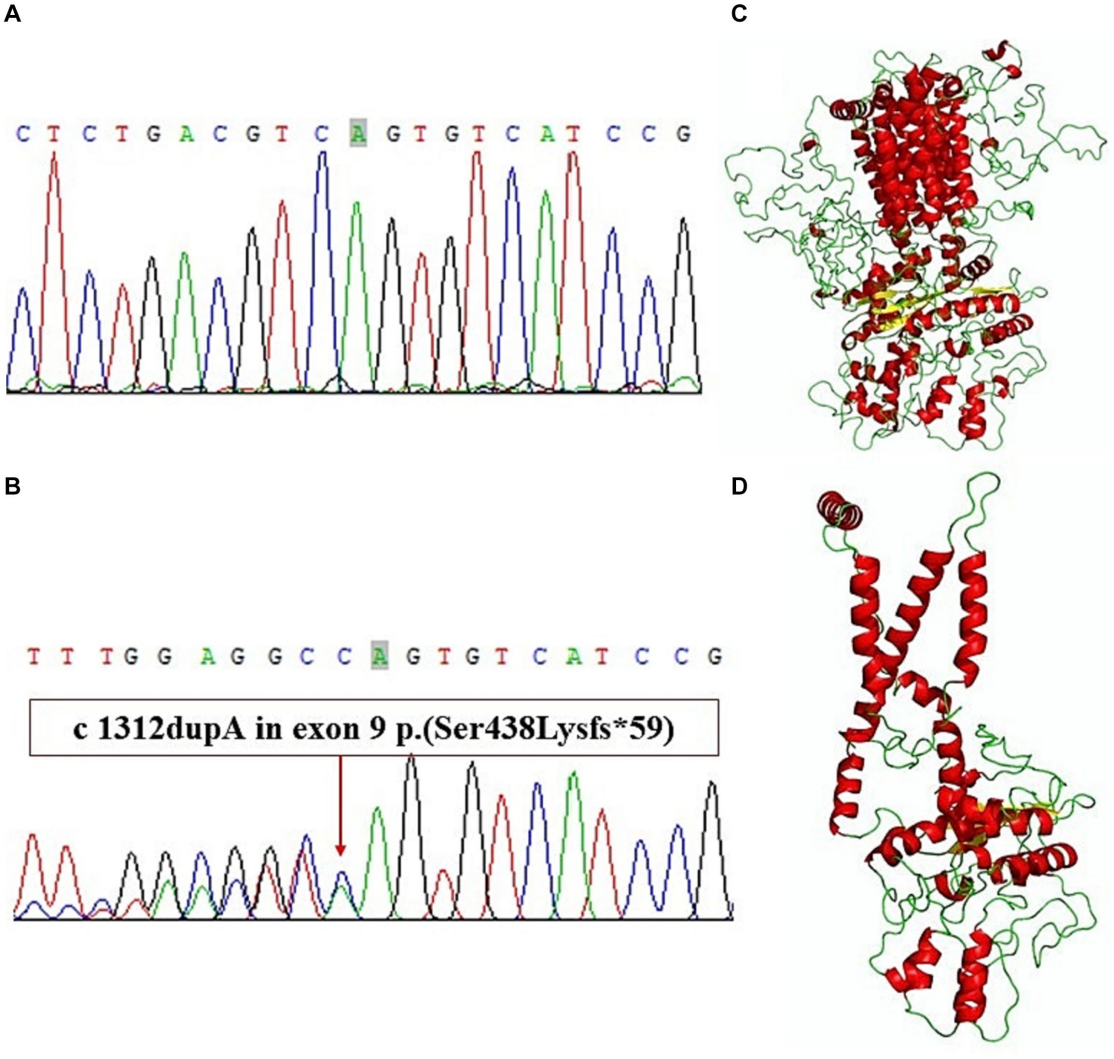
**(A)** Exon 9 of wild-type PTCH1. **(B)** A frameshift mutation in exon 9 of PTCH1. **(C)** Wild-type PTCH1 protein structure models. **(D)** Mutant PTCH1 protein structure models.

We extracted genomic DNA from blood samples of the index case and healthy volunteers because other family members declined to provide blood samples. A gene panel consisting of the exons of 541 genes of monogenic hereditary diseases was used to analyze the genomic DNA of the index case. We obtained genetic DNA through TIANamp Blood DNA kit (The centrifugal type column; DP348); The purity index of genomic DNA samples was 260/280 1.85, 260/230 1.92. The probes used for gene amplification are DNA probes. We get genomic DNA by a first generation sequencing and a second generation sequencing. By verifying that the two sequencing results were identical, so there is no repeat for sequencing experiments. The original data were sequenced by Illumina CASAVA1.8 (Illumina, San Diego, CA, USA), and the reads were compared with the GRCh39/hg17 human genome reference. The results of gene panel sequencing showed a frameshift mutation in exon 9 of PTCH1 (Figures 3A,B) (NM_000264: c.1312dupA:p.Ser438Lysfs). This variant has not been reported according to the ClinVar database. The results of Sanger sequencing confirmed the mutation of the index case, showing a heterozygous state. A three-dimensional structure was modeled to illustrate the effect of the mutation on protein structure according to Protein Data Bank.^1^ Wild-typeand mutant PTCH1 protein structure models were constructed and visualized using I-Tasser^2^ and PyMol software (The PyMOL Molecular Graphics System, versions 2.0, Schrödinger, LLC). The spatial structure of the PTCH1 mutant was significantly different from that of the wild-type PTCH1 (Figures 3C,D).

## Discussion

NBCCS is a rare autosomal dominant disease characterized by developmental abnormalities and tumorigenesis. The PTCH1 gene has been mapped to chromosome 9q22.3-q31 and consists of 24 exons spanning approximately 50 kb that encodes the protein patched homolog 1 (5). PTCH1 contains a transmembrane domain with 12 transmembrane helices. Five transmembrane helices form sterol sensing domains. It also contains two large extracellular domains (ECD1 and ECD2): PTCH1 ECD1 is inserted between transmembrane helices 1 and 2, and PTCH1 ECD2 is inserted between transmembrane helices 7 and 8 (6). Under normal circumstances, PTCH1 inhibits the activity of Smo protein, thereby inhibiting the hedgehog pathway and inhibiting the transcription of downstream target genes, which participate in the growth and development of tissues or organs (3, 7–9).

In the present case, we suspected NBCCS based on the patient’s clinical data and family history. The diagnosis was confirmed by sequencing, which revealed a novel mutation in the PTCH1 gene, c.1312 dupA:p.Ser438Lysfs, that caused a premature stop codon. The c.1312 dupA frameshift variation may result in the production of a truncated protein (p. ser438lysfs) lacking complete transmembrane domains in which both the intracellular and extracellular rings of ECD2 are deleted. This mutant structure will relieve the inhibitory effect on SMO, SMO into human cells and affect downstream genes, activate the transcription factor Glil protein in the Hedgehog signaling pathway, stimulate the continuous expression of various target genes, and a large number of cells with non-programmed unlimited proliferation, inducing tumors, cysts or developmental defects (10). The different regular transcripts may activate the nonsense-mediated mRNA decay (NMD) mechanism, resulting in the degradation of mRNA without turning over an abnormal protein. It may also lead to a decrease in the expression of target proteins and the appearance of various phenotypes (8). According to The American College of Medical Genetics and Genomics (ACMG) guidance for the interpretation of sequence variants. This patient contained a very strong PVS1, a moderate PM6, and a supportive PP4. Therefore, the patient symptoms are related with the mutation (11).

Based on the above information, a molecular genetic study was necessary to confirm the diagnosis of NBCCS. Molecular genetic testing is helpful to confirm the diagnosis in patients with an atypical phenotype or for prenatal diagnosis. Additionally, analysis of the PTCH1 gene in NBCCS provides important information not only for genetic counseling, but also for further research of the correlation between the PTCH1 genotype and the NBCCS phenotype. We recommend that any patient with multiple jaw cysts and/or numerous basal cell carcinomas should be evaluated and undergo genetic testing by a dermatologist to consider a possible diagnosis of Gorlin syndrome.

## Data availability statement

The datasets presented in this study can be found in online repositories. The names of the repository/repositories and accession number(s) can be found at: http://www.wwpdb.org/, NM_000264: c.1312dupA:p.Ser438Lysfs.

## Ethics statement

The studies involving humans were approved by First Hospital of Shanxi Medical University. The studies were conducted in accordance with the local legislation and institutional requirements. The human samples used in this study were acquired from a by-product of routine care or industry. Written informed consent for participation was not required from the participants or the participants’ legal guardians/next of kin in accordance with the national legislation and institutional requirements. Written informed consent was obtained from the individual(s) for the publication of any potentially identifiable images or data included in this article.

## Author contributions

XL: Writing – original draft. TW: Resources, Writing – review & editing. SG: Writing – review & editing. YD: Writing – review & editing. YZ: Writing – review & editing. HL: Writing – review & editing. HH: Writing – review & editing. LL: Writing – review & editing. HY: Writing – review & editing. TH: Writing – review & editing. QW: Writing – review & editing. SQ: Writing – review & editing. RC: Writing – review & editing. XY: Writing – review & editing. HC: Writing – review & editing.

## Footnotes

^1^https://www.rcsb.org/

^2^https://zhanglab.ccmb.med.umich.edu/I-TASSER

## References

[1] Gorlin RJ. Nevoid basal cell carcinoma syndrome. Dermatol Clin. (1995) 13:113–25. doi: 10.1016/S0733-8635(18)30114-1 7712637

[2] Chen JJ Sartori J Aakalu VK Setabutr P. Review of ocular manifestationsof nevoid basal cell carcinoma syndrome: what an ophthalmologist needs to know. Middle East Afr J Ophthalmol. (2015) 22:421–7. doi: 10.4103/0974-9233.167815, PMID: 26692711 PMC4660526

[3] Stone DM Hynes M Armanini M Swanson TA Gu Q Johnson RL. The tumour-suppressor gene patched encodes a candidate receptor for sonic hedgehog. Nature. (1996) 384:129–34. doi: 10.1038/384129a0, PMID: 8906787

[4] Gorlin RJ. Nevoid basal cell carcinoma (Gorlin) syndrome. Genet Med. (2004) 6:530–9. doi: 10.1097/01.gim.0000144188.15902.c4 15545751

[5] Rudolf AF Kinnebrew M Kowatsch C Ansell TB El Omari K Bishop B. The morphogen sonic hedgehog inhibits its receptor patched by a pincer grasp mechanism. Nat Chem Biol. (2019) 15:975–82. doi: 10.1038/s41589-019-0370-y, PMID: 31548691 PMC6764859

[6] Barraud S Delemer B Poirsier-Violle C Bouligand J Mérol JC Grange F. Congenital hypogonadotropic hypogonadism with anosmia and gorlin features caused by a PTCH1 mutation reveals a new candidate gene for Kallmann syndrome. Neuroendocrinology. (2021) 111:99–114. doi: 10.1159/000506640, PMID: 32074614

[7] Wicking C Shanley S Smyth I Gillies S Negus K Graham S. Most germ-line mutations in the nevoid basal cell carcinoma syndrome lead to a premature termination of the PATCHED protein, and no genotype-phenotype correlations are evident. Am J Hum Genet. (1997) 60:21–6. PMID: 8981943. PMID: 8981943 PMC1712561

[8] Johnson RL Rothman AL Xie J Goodrich LV Bare JW Bonifas JM. Human homolog of patched, a candidate gene for the basal cell nevus syndrome. Science. (1996) 272:1668–71. doi: 10.1126/science.272.5268.1668, PMID: 8658145

[9] Chenevix-Trench G Wicking C Berkman J Sharpe H Hockey A Haan E. Further localization of the gene for nevoid basal cell carcinoma syndrome (NBCCS) in 15 Australasian families: linkage and loss of heterozygosity. Am J Hum Genet. (1993) 53:760–7. PMID: 8352281. PMID: 8352281 PMC1682420

[10] Ely LK Truong M. The role of opportunistic bilateral salpingectomy vs tubal occlusion or ligation for ovarian cancer prophylaxis. J Minim Invasive Gynecol. (2017) 24:371–8. doi: 10.1016/j.jmig.2017.01.001 28087480

[11] Richards S Aziz N Bale S Bick D Das S Gastier-Foster J. Standards and guidelines for the interpretation of sequence variants: a joint consensus recommendation of the American College of Medical Genetics and Genomics and the Association for Molecular Pathology. Genet Med. (2015) 17:405–24. doi: 10.1038/gim.2015.30, PMID: 25741868 PMC4544753

