# Corrigendum: Case report: A novel PTCH1 frameshift mutation leading to nevoid basal cell carcinoma syndrome

**DOI:** 10.3389/fmed.2025.1574170

**Published:** 2025-03-28

**Authors:** Xiaoqing Lang, Ting Wang, Shuping Guo, Yao Dang, Yingjie Zhang, Hongye Liu, Hongxia He, Li Li, Huajie Yuan, Ting He, Qiong Wang, Shiyu Qin, Runping Cheng, Xingquan Yan, Hongzhou Cui

**Affiliations:** ^1^Department of Dermatology, First Hospital of Shanxi Medical University, Taiyuan, China; ^2^Department of Dermatology, Shanxi Provincial Integrated Traditional Chinese Medicine and Western Medicine Hospital, Taiyuan, China; ^3^Department of Nursing, Fenyang College of Shanxi Medical University, Fenyang, China

**Keywords:** novel, frameshift mutation, nevoid basal cell carcinoma syndrome, PTCH1, case report

In the published article, there was an error in affiliation number 2. Instead of “Department of Dermatology, The Affiliated Hospital of Shanxi University of Traditional Chinese Medicine, Taiyuan, China”, it should be “Department of Dermatology, Shanxi Provincial Integrated Traditional Chinese Medicine and Western Medicine Hospital, Taiyuan, China.”

The authors apologize for this error and state that this does not change the scientific conclusions of the article in any way. The original article has been updated.

