# Supplementary material for: Case report: A novel PTCH1 frameshift mutation leading to nevoid basal cell carcinoma syndrome
Source: Front Med (Lausanne). 2024 Mar 4;11:1327505. doi: 10.3389/fmed.2024.1327505 (PMC10946671; doi:10.3389/fmed.2024.1327505)
Supplement: Supplementary file 1 [file Table_1.docx]

Comparison of wild type and mutant amino acid mutations

Mutant 1 MASAGNAAEPQDRGGGGSGCIGAPGRPAGGGRRRRTGGLRRAAAPDRDYLHRPSYCDAAF 60

||||||||||||||||||||||||||||||||||||||||||||||||||||||||||||

Wild-type 1 MASAGNAAEPQDRGGGGSGCIGAPGRPAGGGRRRRTGGLRRAAAPDRDYLHRPSYCDAAF 60

Mutant 61 ALEQISKGKATGRKAPLWLRAKFQRLLFKLGCYIQKNCGKFLVVGLLIFGAFAVGLKAAN 120

||||||||||||||||||||||||||||||||||||||||||||||||||||||||||||

Wild-type 61 ALEQISKGKATGRKAPLWLRAKFQRLLFKLGCYIQKNCGKFLVVGLLIFGAFAVGLKAAN 120

Mutant 121 LETNVEELWVEVGGRVSRELNYTRQKIGEEAMFNPQLMIQTPKEEGANVLTTEALLQHLD 180

||||||||||||||||||||||||||||||||||||||||||||||||||||||||||||

Wild-type 121 LETNVEELWVEVGGRVSRELNYTRQKIGEEAMFNPQLMIQTPKEEGANVLTTEALLQHLD 180

Mutant 181 SALQASRVHVYMYNRQWKLEHLCYKSGELITETGYMDQIIEYLYPCLIITPLDCFWEGAK 240

||||||||||||||||||||||||||||||||||||||||||||||||||||||||||||

Wild-type 181 GCTCTGGAGCAGATTTCCAAGGGGAAGGCTACTGGCCGGAAAGCGCCGCTGTGGCTGAGA 240

Mutant 241 LQSGTAYLLGKPPLRWTNFDPLEFLEELKKINYQVDSWEEMLNKAEVGHGYMDRPCLNPA 300

||||||||||||||||||||||||||||||||||||||||||||||||||||||||||||

Wild-type 241 LQSGTAYLLGKPPLRWTNFDPLEFLEELKKINYQVDSWEEMLNKAEVGHGYMDRPCLNPA 300

Mutant 301 DPDCPATAPNKNSTKPLDMALVLNGGCHGLSRKYMHWQEELIVGGTVKNSTGKLVSAHAL 360

||||||||||||||||||||||||||||||||||||||||||||||||||||||||||||

Wild-type 301 DPDCPATAPNKNSTKPLDMALVLNGGCHGLSRKYMHWQEELIVGGTVKNSTGKLVSAHAL 360

Mutant 361 QTMFQLMTPKQMYEHFKGYEYVSHINWNEDKAAAILEAWQRTYVEVVHQSVAQNSTQKVL 420

||||||||||||||||||||||||||||||||||||||||||||||||||||||||||||

Wild-type 361 QTMFQLMTPKQMYEHFKGYEYVSHINWNEDKAAAILEAWQRTYVEVVHQSVAQNSTQKVL 420

Mutant 421 SFTTTTLDDILKSFSDVKCHPRGQRLLTHARLCLSNHAALGLLQVPGCRGAGWRPAGCTV 480

||||||||||||||||||||||||||||||||||||||||||||||||||||||||||||

Wild-type 421 SFTTTTLDDILKSFSDVSVIRVASGYLLMLAYACLTMLRWDCSKSQGAVGLAGVLLVALS 480

Mutant 481 SGCRTGPVLIDRNFL* 495

||||||||||||||||

Wild-type 481 VAAGLGLCSLIGISFNAATTQVLPFLALGVGVDDVFLLAHAFSETGQNKRIPFEDRTGEC 540

Wild-type 541 LKRTGASVALTSISNVTAFFMAALIPIPALRAFSLQAAVVVVFNFAMVLLIFPAILSMDL 600

Wild-type 601 YRREDRRLDIFCCFTSPCVSRVIQVEPQAYTDTHDNTRYSPPPPYSSHSFAHETQITMQS 660

Wild-type 661 TVQLRTEYDPHTHVYYTTAEPRSEISVQPVTVTQDTLSCQSPESTSSTRDLLSQFSDSSL 720

Wild-type 721 HCLEPPCTKWTLSSFAEKHYAPFLLKPKAKVVVIFLFLGLLGVSLYGTTRVRDGLDLTDI 780

Wild-type 781 VPRETREYDFIAAQFKYFSFYNMYIVTQKADYPNIQHLLYDLHRSFSNVKYVMLEENKQL 840

Wild-type 841 PKMWLHYFRDWLQGLQDAFDSDWETGKIMPNNYKNGSDDGVLAYKLLVQTGSRDKPIDIS 900

Wild-type 901 QLTKQRLVDADGIINPSAFYIYLTAWVSNDPVAYAASQANIRPHRPEWVHDKADYMPETR 960

Wild-type 961 LRIPAAEPIEYAQFPFYLNGLRDTSDFVEAIEKVRTICSNYTSLGLSSYPNGYPFLFWEQ 1020

Wild-type1021 YIGLRHWLLLFISVVLACTFLVCAVFLLNPWTAGIIVMVLALMTVELFGMMGLIGIKLSA 1080

Wild-type1081 VPVVILIASVGIGVEFTVHVALAFLTAIGDKNRRAVLALEHMFAPVLDGAVSTLLGVLML 1140

Wild-type1141 AGSEFDFIVRYFFAVLAILTILGVLNGLVLLPVLLSFFGPYPEVSPANGLNRLPTPSPEP 1200

Wild-type1201 PPSVVRFAMPPGHTHSGSDSSDSEYSSQTTVSGLSEELRHYEAQQGAGGPAHQVIVEATE 1260

Wild-type1261 NPVFAHSTVVHPESRHHPPSNPRQQPHLDSGSLPPGRQGQQPRRDPPREGLWPPPYRPRR 1319

Wild-type1320 DAFEISTEGHSGPSNRARWGPRGARSHNPRNPASTAMGSSVPGYCQPITTVTASASVTVA 1379

Wild-type1380 VHPPPVPGPGRNPRGGLCPGYPETDHGLFEDPHVPFHVRCERRDSKVEVIELQDVECEER 1439

Wild-type1440 PRGSSSN* 1447
