# Supplementary material for: Case report: A novel PTCH1 frameshift mutation leading to nevoid basal cell carcinoma syndrome
Source: Front Med (Lausanne). 2024 Mar 4;11:1327505. doi: 10.3389/fmed.2024.1327505 (PMC10946671; doi:10.3389/fmed.2024.1327505)
Supplement: Supplementary file 2 [file Table_2.docx]

Comparison of exon mutations between wild-type and mutant

RID: PPKY61UR11R

Job Title:Nucleotide Sequence

Program: BLASTN

Wild-type:None ID: lcl|Mutant_441179(dna) Length: 4344

Mutant #1: Mutant ID: lcl|Mutant_441177 Length: 4345

Sequences producing significant alignments:

Scientific Common Max Total Mutant E Per. Acc.

Description Name Name Taxid Score Score cover Value Ident Len Accession

None provided 0 8017 8017 100% 0.0 99.98 4344 Mutant_441179

Alignments:

>

Sequence ID: Query_441179 Length: 4344

Range 1: 1 to 4344

Score:8017 bits(4341), Expect:0.0,

Identities:4344/4345(99%), Gaps:1/4345(0%), Strand: Plus/Plus

Mutant 1 ATGGCCTCGGCTGGTAACGCCGCCGAGCCCCAGGACCGCGGCGGCGGCGGCAGCGGCTGT 60

||||||||||||||||||||||||||||||||||||||||||||||||||||||||||||

Wild-type 1 ATGGCCTCGGCTGGTAACGCCGCCGAGCCCCAGGACCGCGGCGGCGGCGGCAGCGGCTGT 60

Mutant 61 ATCGGTGCCCCGGGACGGCCGGCTGGAGGCGGGAGGCGCAGACGGACgggggggCTGCGC 120

||||||||||||||||||||||||||||||||||||||||||||||||||||||||||||

Wild-type 61 ATCGGTGCCCCGGGACGGCCGGCTGGAGGCGGGAGGCGCAGACGGACGGGGGGGCTGCGC 120

Mutant 121 CGTGCTGCCGCGCCGGACCGGGACTATCTGCACCGGCCCAGCTACTGCGACGCCGCCTTC 180

||||||||||||||||||||||||||||||||||||||||||||||||||||||||||||

Wild-type 121 CGTGCTGCCGCGCCGGACCGGGACTATCTGCACCGGCCCAGCTACTGCGACGCCGCCTTC 180

Mutant 181 GCTCTGGAGCAGATTTCCAAGGGGAAGGCTACTGGCCGGAAAGCGCCGCTGTGGCTGAGA 240

||||||||||||||||||||||||||||||||||||||||||||||||||||||||||||

Wild-type 181 GCTCTGGAGCAGATTTCCAAGGGGAAGGCTACTGGCCGGAAAGCGCCGCTGTGGCTGAGA 240

Mutant 241 GCGAAGTTTCAGAGACTCTTATTTAAACTGGGTTGTTACATTCaaaaaaaCTGCGGCAAG 300

||||||||||||||||||||||||||||||||||||||||||||||||||||||||||||

Wild-type 241 GCGAAGTTTCAGAGACTCTTATTTAAACTGGGTTGTTACATTCAAAAAAACTGCGGCAAG 300

Mutant 301 TTCTTGGTTGTGGGCCTCCTCATATTTGGGGCCTTCGCGGTGGGATTAAAAGCAGCGAAC 360

||||||||||||||||||||||||||||||||||||||||||||||||||||||||||||

Wild-type 301 TTCTTGGTTGTGGGCCTCCTCATATTTGGGGCCTTCGCGGTGGGATTAAAAGCAGCGAAC 360

Mutant 361 CTCGAGACCAACGTGGAGGAGCTGTGGGTGGAAGTTGGAGGACGAGTAAGTCGTGAATTA 420

||||||||||||||||||||||||||||||||||||||||||||||||||||||||||||

Wild-type 361 CTCGAGACCAACGTGGAGGAGCTGTGGGTGGAAGTTGGAGGACGAGTAAGTCGTGAATTA 420

Mutant 421 AATTATACTCGCCAGAAGATTGGAGAAGAGGCTATGTTTAATCCTCAACTCATGATACAG 480

||||||||||||||||||||||||||||||||||||||||||||||||||||||||||||

Wild-type 421 AATTATACTCGCCAGAAGATTGGAGAAGAGGCTATGTTTAATCCTCAACTCATGATACAG 480

Mutant 481 ACCCCTAAAGAAGAAGGTGCTAATGTCCTGACCACAGAAGCGCTCCTACAACACCTGGAC 540

||||||||||||||||||||||||||||||||||||||||||||||||||||||||||||

Wild-type 481 ACCCCTAAAGAAGAAGGTGCTAATGTCCTGACCACAGAAGCGCTCCTACAACACCTGGAC 540

Mutant 541 TCGGCACTCCAGGCCAGCCGTGTCCATGTATACATGTACAACAGGCAGTGGAAATTGGAA 600

||||||||||||||||||||||||||||||||||||||||||||||||||||||||||||

Wild-type 541 TCGGCACTCCAGGCCAGCCGTGTCCATGTATACATGTACAACAGGCAGTGGAAATTGGAA 600

Mutant 601 CATTTGTGTTACAAATCAGGAGAGCTTATCACAGAAACAGGTTACATGGATCAGATAATA 660

||||||||||||||||||||||||||||||||||||||||||||||||||||||||||||

Wild-type 601 CATTTGTGTTACAAATCAGGAGAGCTTATCACAGAAACAGGTTACATGGATCAGATAATA 660

Mutant 661 GAATATCTTTACCCTTGTTTGATTATTACACCTTTGGACTGCTTCTGGGAAGGGGCGAAA 720

||||||||||||||||||||||||||||||||||||||||||||||||||||||||||||

Wild-type 661 GAATATCTTTACCCTTGTTTGATTATTACACCTTTGGACTGCTTCTGGGAAGGGGCGAAA 720

Mutant 721 TTACAGTCTGGGACAGCATACCTCCTAGGTAAACCTCCTTTGCGGTGGACAAACTTCGAC 780

||||||||||||||||||||||||||||||||||||||||||||||||||||||||||||

Wild-type 721 TTACAGTCTGGGACAGCATACCTCCTAGGTAAACCTCCTTTGCGGTGGACAAACTTCGAC 780

Mutant 781 CCTTTGGAATTCCTGGAAGAGTTAAAGAAAATAAACTATCAAGTGGACAGCTGGGAGGAA 840

||||||||||||||||||||||||||||||||||||||||||||||||||||||||||||

Wild-type 781 CCTTTGGAATTCCTGGAAGAGTTAAAGAAAATAAACTATCAAGTGGACAGCTGGGAGGAA 840

Mutant 841 ATGCTGAATAAGGCTGAGGTTGGTCATGGTTACATGGACCGCCCCTGCCTCAATCCGGCC 900

||||||||||||||||||||||||||||||||||||||||||||||||||||||||||||

Wild-type 841 ATGCTGAATAAGGCTGAGGTTGGTCATGGTTACATGGACCGCCCCTGCCTCAATCCGGCC 900

Mutant 901 GATCCAGACTGCCCCGCCACAGCCCCCAACAAAAATTCAACCAAACCTCTTGATATGGCC 960

||||||||||||||||||||||||||||||||||||||||||||||||||||||||||||

Wild-type 901 GATCCAGACTGCCCCGCCACAGCCCCCAACAAAAATTCAACCAAACCTCTTGATATGGCC 960

Mutant 961 CTTGTTTTGAATGGTGGATGTCATGGCTTATCCAGAAAGTATATGCACTGGCAGGAGGAG 1020

||||||||||||||||||||||||||||||||||||||||||||||||||||||||||||

Wild-type 961 CTTGTTTTGAATGGTGGATGTCATGGCTTATCCAGAAAGTATATGCACTGGCAGGAGGAG 1020

Mutant 1021 TTGATTGTGGGTGGCACAGTCAAGAACAGCACTGGAAAACTCGTCAGCGCCCATGCCCTG 1080

||||||||||||||||||||||||||||||||||||||||||||||||||||||||||||

Wild-type1021 TTGATTGTGGGTGGCACAGTCAAGAACAGCACTGGAAAACTCGTCAGCGCCCATGCCCTG 1080

Mutant 1081 CAGACCATGTTCCAGTTAATGACTCCCAAGCAAATGTACGAGCACTTCAAGGGGTACGAG 1140

||||||||||||||||||||||||||||||||||||||||||||||||||||||||||||

Wild-type1081 CAGACCATGTTCCAGTTAATGACTCCCAAGCAAATGTACGAGCACTTCAAGGGGTACGAG 1140

Mutant 1141 TATGTCTCACACATCAACTGGAACGAGGACAAAGCGGCAGCCATCCTGGAGGCCTGGCAG 1200

||||||||||||||||||||||||||||||||||||||||||||||||||||||||||||

Wild-type1141 TATGTCTCACACATCAACTGGAACGAGGACAAAGCGGCAGCCATCCTGGAGGCCTGGCAG 1200

Mutant 1201 AGGACATATGTGGAGGTGGTTCATCAGAGTGTCGCACAGAACTCCACTCAAAAGGTGCTT 1260

||||||||||||||||||||||||||||||||||||||||||||||||||||||||||||

Wild-type1201 AGGACATATGTGGAGGTGGTTCATCAGAGTGTCGCACAGAACTCCACTCAAAAGGTGCTT 1260

Mutant 1261 TCCTTCACCACCACGACCCTGGACGACATCCTGAAATCCTTCTCTGACGTCAAGTGTCAT 1320

||||||||||||||||||||||||||||||||||||||||||||||||||| ||||||||

Wild-type1261 TCCTTCACCACCACGACCCTGGACGACATCCTGAAATCCTTCTCTGACGTC-AGTGTCAT 1319

Mutant 1321 CCGCGTGGCCAGCGGCTACTTACTCATGCTCGCCTATGCCTGTCTAACCATGCTGCGCTG 1380

||||||||||||||||||||||||||||||||||||||||||||||||||||||||||||

Wild-type1320 CCGCGTGGCCAGCGGCTACTTACTCATGCTCGCCTATGCCTGTCTAACCATGCTGCGCTG 1379

Mutant 1381 GGACTGCTCCAAGTCCCAGGGTGCCGTGGGGCTGGCTGGCGTCCTGCTGGTTGCACTGTC 1440

||||||||||||||||||||||||||||||||||||||||||||||||||||||||||||

Wild-type1380 GGACTGCTCCAAGTCCCAGGGTGCCGTGGGGCTGGCTGGCGTCCTGCTGGTTGCACTGTC 1439

Mutant 1441 AGTGGCTGCAGGACTGGGCCTGTGCTCATTGATCGGAATTTCCTTTAA 1500

||||||||||||||||||||||||||||||||||||||||||||||||

Wild-type1440 AGTGGCTGCAGGACTGGGCCTGTGCTCATTGATCGGAATTTCCTTTAACGCTGCAACAAC 1499

Wild-type1500 TCAGGTTTTGCCATTTCTCGCTCTTGGTGTTGGTGTGGATGATGTTTTTCTTCTGGCCCA 1559

Wild-type1560 CGCCTTCAGTGAAACAGGACAGAATAAAAGAATCCCTTTTGAGGACAGGACCGGGGAGTG 1619

Wild-type1620 CCTGAAGCGCACAGGAGCCAGCGTGGCCCTCACGTCCATCAGCAATGTCACAGCCTTCTT 1679

Wild-type1680 CATGGCCGCGTTAATCCCAATTCCCGCTCTGCGGGCGTTCTCCCTCCAGGCAGCGGTAGT 1739

Wild-type1740 AGTGGTGTTCAATTTTGCCATGGTTCTGCTCATTTTTCCTGCAATTCTCAGCATGGATTT 1799

Wild-type1800 ATATCGACGCGAGGACAGGAGACTGGATATTTTCTGCTGTTTTACAAGCCCCTGCGTCAG 1859

Wild-type1860 CAGAGTGATTCAGGTTGAACCTCAGGCCTACACCGACACACACGACAATACCCGCTACAG 1919

Wild-type1920 CCCCCCACCTCCCTACAGCAGCCACAGCTTTGCCCATGAAACGCAGATTACCATGCAGTC 1979

Wild-type1980 CACTGTCCAGCTCCGCACGGAGTACGACCCCCACACGCACGTGTACTACACCACCGCTGA 2039

Wild-type2040 GCCGCGCTCCGAGATCTCTGTGCAGCCCGTCACCGTGACACAGGACACCCTCAGCTGCCA 2099

Wild-type2100 GAGCCCAGAGAGCACCAGCTCCACAAGGGACCTGCTCTCCCAGTTCTCCGACTCCAGCCT 2159

Wild-type2160 CCACTGCCTCGAGCCCCCCTGTACGAAGTGGACACTCTCATCTTTTGCTGAGAAGCACTA 2219

Wild-type2220 TGCTCCTTTCCTCTTGAAACCAAAAGCCAAGGTAGTGGTGATCTTCCTTTTTCTGGGCTT 2279

Wild-type2280 GCTGGGGGTCAGCCTTTATGGCACCACCCGAGTGAGAGACGGGCTGGACCTTACGGACAT 2339

Wild-type2340 TGTACCTCGGGAAACCAGAGAATATGACTTTATTGCTGCACAATTCAAATACTTTTCTTT 2399

Wild-type2400 CTACAACATGTATATAGTCACCCAGAAAGCAGACTACCCGAATATCCAGCACTTACTTTA 2459

Wild-type2460 CGACCTACACAGGAGTTTCAGTAACGTGAAGTATGTCATGTTGGAAGAAAACAAACAGCT 2519

Wild-type2520 TCCCAAAATGTGGCTGCACTACTTCAGAGACTGGCTTCAGGGACTTCAGGATGCATTTGA 2579

Wild-type2580 CAGTGACTGGGAAACCGGGAAAATCATGCCAAACAATTACAAGAATGGATCAGACGATGG 2639

Wild-type2640 AGTCCTTGCCTACAAACTCCTGGTGCAAACCGGCAGCCGCGATAAGCCCATCGACATCAG 2699

Wild-type2700 CCAGTTGACTAAACAGCGTCTGGTGGATGCAGATGGCATCATTAATCCCAGCGCTTTCTA 2759

Wild-type2760 CATCTACCTGACGGCTTGGGTCAGCAACGACCCCGTCGCGTATGCTGCCTCCCAGGCCAA 2819

Wild-type2820 CATCCGGCCACACCGACCAGAATGGGTCCACGACAAAGCCGACTACATGCCTGAAACAAG 2879

Wild-type2880 GCTGAGAATCCCGGCAGCAGAGCCCATCGAGTATGCCCAGTTCCCTTTCTACCTCAACGG 2939

Wild-type2940 CTTGCGGGACACCTCAGACTTTGTGGAGGCAATTGAAAAAGTAAGGACCATCTGCAGCAA 2999

Wild-type3000 CTATACGAGCCTGGGGCTGTCCAGTTACCCCAACGGCTACCCCTTCCTCTTCTGGGAGCA 3059

Wild-type3060 GTACATCGGCCTCCGCCACTGGCTGCTGCTGTTCATCAGCGTGGTGTTGGCCTGCACATT 3119

Wild-type3120 CCTCGTGTGCGCTGTCTTCCTTCTGAACCCCTGGACGGCCGGGATCATTGTGATGGTCCT 3179

Wild-type3180 GGCGCTGATGACGGTCGAGCTGTTCGGCATGATGGGCCTCATCGGAATCAAGCTCAGTGC 3239

Wild-type3240 CGTGCCCGTGGTCATCCTGATCGCTTCTGTTGGCATAGGAGTGGAGTTCACCGTTCACGT 3299

Wild-type3300 TGCTTTGGCCTTTCTGACGGCCATCGGCGACAAGAACCGCAGGGCTGTGCTTGCCCTGGA 3359

Wild-type3360 GCACATGTTTGCACCCGTCCTGGATGGCGCCGTGTCCACTCTGCTGGGAGTGCTGATGCT 3419

Wild-type3420 GGCGGGATCTGAGTTCGACTTCATTGTCAGGTATTTCTTTGCTGTGCTGGCGATCCTCAC 3479

Wild-type3480 CATCCTCGGCGTTCTCAATGGGCTGGTTTTGCTTCCCGTGCTTTTGTCTTTCTTTGGACC 3539

Wild-type3540 ATATCCTGAGGTGTCTCCAGCCAACGGCTTGAACCGCCTGCCCACACCCTCCCCTGAGCC 3599

Wild-type3600 ACCCCCCAGCGTGGTCCGCTTCGCCATGCCGCCCGGCCACACGCACAGCGGGTCTGATTC 3659

Wild-type3660 CTCCGACTCGGAGTATAGTTCCCAGACGACAGTGTCAGGCCTCAGCGAGGAGCTTCGGCA 3719

Wild-type3720 CTACGAGGCCCAGCAGGGCGCGGGAGGCCCTGCCCACCAAGTGATCGTGGAAGCCACAGA 3779

Wild-type3780 AAACCCCGTCTTCGCCCACTCCACTGTGGTCCATCCCGAATCCAGGCATCACCCACCCTC 3839

Wild-type3840 GAACCCGAGACAGCAGCCCCACCTGGACTCAGGGTCCCTGCCTCCCGGACGGCAAGGCCA 3899

Wild-type3900 GCAGCCCCGCAGGGACCCCCCCAGAGAAGGCTTGTGGCCACCCCCCTACAGACCGCGCAG 3959

Wild-type3960 AGACGCTTTTGAAATTTCTACTGAAGGGCATTCTGGCCCTAGCAATAGGGCCCGCTGGGG 4019

Wild-type4020 CCCTCGCGGGGCCCGTTCTCACAACCCTCGGAACCCAGCGTCCACTGCCATGGGCAGCTC 4079

Wild-type4080 CGTGCCCGGCTACTGCCAGCCCATCACCACTGTGACGGCTTCTGCCTCCGTGACTGTCGC 4139

Wild-type4140 CGTGCACCCGCCGCCTGTCCCTGGGCCTGGGCGGAACCCCCGAGGGGGACTCTGCCCAGG 4199

Wild-type4200 CTACCCTGAGACTGACCACGGCCTGTTTGAGGACCCCCACGTGCCTTTCCACGTCCGGTG 4259

Wild-type4260 TGAGAGGAGGGATTCGAAGGTGGAAGTCATTGAGCTGCAGGACGTGGAATGCGAGGAGAG 4319

Wild-type4320 GCCCCGGGGAAGCAGCTCCAACTGA 4344
