# Supplementary material for: Case report: A novel PTCH1 frameshift mutation leading to nevoid basal cell carcinoma syndrome
Source: Front Med (Lausanne). 2024 Mar 4;11:1327505. doi: 10.3389/fmed.2024.1327505 (PMC10946671; doi:10.3389/fmed.2024.1327505)
Supplement: Supplementary file 3 [file Table_3.docx]

Primer design for PTCH1

1.PTCH1:NM_000264:exon9:c.1312dupA:p.S438fs

gtggccagcaaatgaacagggggaggagggccataagaatgagttgttttggattagtggggagccgatgggaccgcaagtcaattgtgaagttaacggagaagagacgagcagtctcagccctggagtaaatacagcactcagagtgaactccagcgacaccagccctcagcagcacccactgccactgattagccatggcccttgtcctaaaacagggttctttaatgttagcccaacttcagcttctctccctgccctggaatcacgtagaacttttttaaaaaatgcatcagaaaaaaagaagggctgcgctgtgtcacctgagatctgtgctgtcgaggcttgtggaagtgttcattgcatttgggcatttcgcattctgttgtgaccacaggtggttcatcagagtgtcgcacagaactccactcaaaaggtgctttccttcaccaccacgaccctggacgacatcctgaaatccttctctgacgtcagtgtcatccgcgtggccagcggctacttactcatggtaacgctcgatgccatgctcctgggggctggagtttggtttggttgttttagtctttacttttccatgactgctcctgcttcttaactgctcttaacatcgatgtgcatccaggacagagagagcgtggcttcacagtggtt

## **PTCH1-E09-F/R**

|  | **Sequence (5'->3')** | **Template strand** | **Length** | **Start** | **Stop** | **Tm** | **GC%** | **Self complementarity** | **Self 3' complementarity** |
| --- | --- | --- | --- | --- | --- | --- | --- | --- | --- |
| **Forward primer** | TGTTTTGGATTAGTGGGGAG | Plus | 20 | 213 | 232 | 54.93 | 45.00 | 2.00 | 0.00 |
| **Reverse primer** | AAGAGCAGTTAAGAAGCAGG | Minus | 20 | 792 | 773 | 55.37 | 45.00 | 4.00 | 0.00 |
| **Product length** | 580 | | | | | | | | |

**Products on intended targets**

>[NC_000009.12](https://www.ncbi.nlm.nih.gov/nucleotide/568815589?from=95477960&to=95478539&report=gbwithparts) Homo sapiens chromosome 9, GRCh38.p13 Primary Assembly

product length = 580

Features associated with this product:

[protein patched homolog 1 isoform s](https://www.ncbi.nlm.nih.gov/nucleotide/568815589?from=95446912&to=95485815&report=gbwithparts)

[protein patched homolog 1 isoform s](https://www.ncbi.nlm.nih.gov/nucleotide/568815589?from=95446912&to=95485815&report=gbwithparts)

Forward primer 1 TGTTTTGGATTAGTGGGGAG 20

Template 95478539 .................... 95478520

Reverse primer 1 AAGAGCAGTTAAGAAGCAGG 20

Template 95477960 .................... 95477979
